# Identification of a prognostic signature based on immunogenic adverse event-related genes to guide therapy for non-small cell lung cancer

**DOI:** 10.3389/fimmu.2025.1656375

**Published:** 2026-01-12

**Authors:** Jun Zhu, Qing Ye, Gang Li, Lidong Liu, Shushu Tian, Shuangyan Li, Maoru Wen, Luying Shen, Jiang Wang, Xinmiao Song, Hong Chen, Yi Li

**Affiliations:** 1 Kunming Medical University, Kunming, Yunnan, China; 2 th Department of Oncology, 920 Hospital of Joint Logistics Support Force, Kunming, Yunnan, China; 3 Department of Radiation Oncology, The First Affiliated Hospital of Kunming Medical University, Kunming, Yunnan, China; 4 Department of Radiation Oncology, Eye, Ear, Nose, and Throat Hospital, Fudan University, Shanghai, China

**Keywords:** immune-related adverse events, immunotherapy, non-small cell lung cancer, prognosis, risk score

## Abstract

**Background:**

Immune checkpoint inhibitors (ICIs) improve outcomes in non−small cell lung cancer (NSCLC), yet reliable predictive biomarkers are still lacking. Given that immune−related adverse events (irAEs) often correlate with better ICI efficacy, this study aimed to develop and validate an irAE−related gene signature for risk stratification and treatment–response prediction in NSCLC.

**Methods:**

Transcriptomic and clinical data were obtained from the Cancer Genome Atlas (TCGA) and Gene Expression Omnibus (GEO) databases, designated as training and validation cohorts, respectively. Least absolute shrinkage and selection operator (LASSO) regression and Cox proportional hazards models were applied to identify eight irAE-associated genes for constructing a risk score (RS). Multivariate analyses evaluated differences in overall survival (OS), immunotherapy response, irAE incidence, immune escape potential, and tumor microenvironment (TME) profiles between high- and low-risk groups. Prognostic performance was validated using Kaplan–Meier curves, Cox regression, and receiver operating characteristic (ROC) analysis. A nomogram integrating RS with clinical factors was developed to improve prediction stability.

**Results:**

Via gene set variation analysis (GSVA) enrichment of 30 known immunologic gene sets in TCGA-LC, we stratified samples by immune score (cutoff = 0.23), identified 1,057 differentially expressed genes (DEGs) between groups, intersected with cancer-normal DEGs, and selected 132 irAE-related DEGs via Pearson correlation—genes functionally tied to T-cell activation and cytokine-mediated signaling pathways. LASSO–Cox regression derived an eight-gene prognostic signature demonstrating robust cross-cohort validation [area under the curve (AUC): TCGA = 0.770, GSE50081 = 0.767, GSE37745 = 0.758] and predictive accuracy for irAEs in GSE186143 (AUC = 0.807). In a validation NSCLC cohort (*n* = 41), irAE occurrence correlated with superior clinical outcomes: objective response rate (87.50% *vs*. 44.00%), disease control rate (93.75% *vs*. 76.00%), median progression-free survival (20.0 *vs*. 9.9 months), and overall survival (21.4 *vs*. 12.1 months). Three key risk genes (POU2AF1, ANKRD44, CRTAM) showed significantly elevated protein expression levels and positive rates in irAE patients. High RS cohorts exhibited immunosuppressive TME characteristics and increased immune escape potential. A clinically relevant nomogram integrating RS with clinical factors (age, stage, and tumor recurrence) improved prognostic stability.

**Conclusions:**

Our irAE-associated gene signature robustly stratifies NSCLC patients for immunotherapy response and survival. Integrating RS with clinical parameters provides a practical tool to balance efficacy and safety.

## 1 Introduction

Lung cancer is the most common malignant tumor with the highest mortality globally, and over 87% of patients have non-small cell lung cancer (NSCLC) (1). In recent years, immunotherapy designed to induce a cellular immune response has become a mainstay of cancer therapy. Specifically, immune checkpoint inhibitors (ICIs), targeting the programmed cell death protein-1/programmed cell death ligand-1 (PD-1/PD-L1) axis, have been approved for first- or second-line treatments of NSCLC, regardless of the patient’s history or driver mutational status (2, 3). As compared with conventional chemotherapy, immunotherapy can achieve unprecedented and sustained responses in patients with advanced cancer (4, 5). However, the efficacy of ICIs is of remarkable diversity, with an objective response rate of less than 30% in patients with advanced NSCLC (6). Therefore, there is an urgent clinical need for novel tools to predict therapeutic efficacy, which could guide clinical treatment decisions more effectively.

The widespread use of ICIs is often accompanied by an increased risk of immune-related adverse events (irAEs) (7, 8). The incidence of irAEs in patients treated with ICIs reaches up to 70%, which can diminish quality of life, increase healthcare costs, and even lead to severe toxic effects (9, 10). However, due to the immune system activation and inflammatory response induced by ICIs, irAEs have been reported to correlate with improved survival in cancer patients treated with ICIs (11, 12). For example, cutaneous irAEs have been identified as an independent prognostic factor for progression-free survival (PFS) in melanoma patients treated with nivolumab (13). Similarly, patients with irAEs have shown increased PFS and higher objective response rate (ORR) compared to those without irAEs in NSCLC (14). Studies further revealed that the occurrence of irAEs, such as rash, was closely relevant to the efficacy of ICIs in hepatocellular carcinoma patients (15). Recently, advances in genome sequencing technologies have provided new approaches to elucidate the underlying cancer gene/pathway-associated molecular mechanisms, facilitating the identification of both tumor intrinsic and extrinsic factors as predictive biomarkers. Therefore, focusing on molecular characteristics of irAEs could offer important insights into precision medicine for NSCLC patients.

In the present study, we constructed a risk signature of eight genes crucially associated with irAEs. We evaluated the differences in overall survival (OS), immunotherapy response, irAEs, immune escape, and tumor microenvironment (TME) between high- and low-risk score (RS) groups. Finally, we developed a nomogram model to improve the utility of RS.

## 2 Materials and methods

### 2.1 Study design and data acquisition

**Figure 1:**
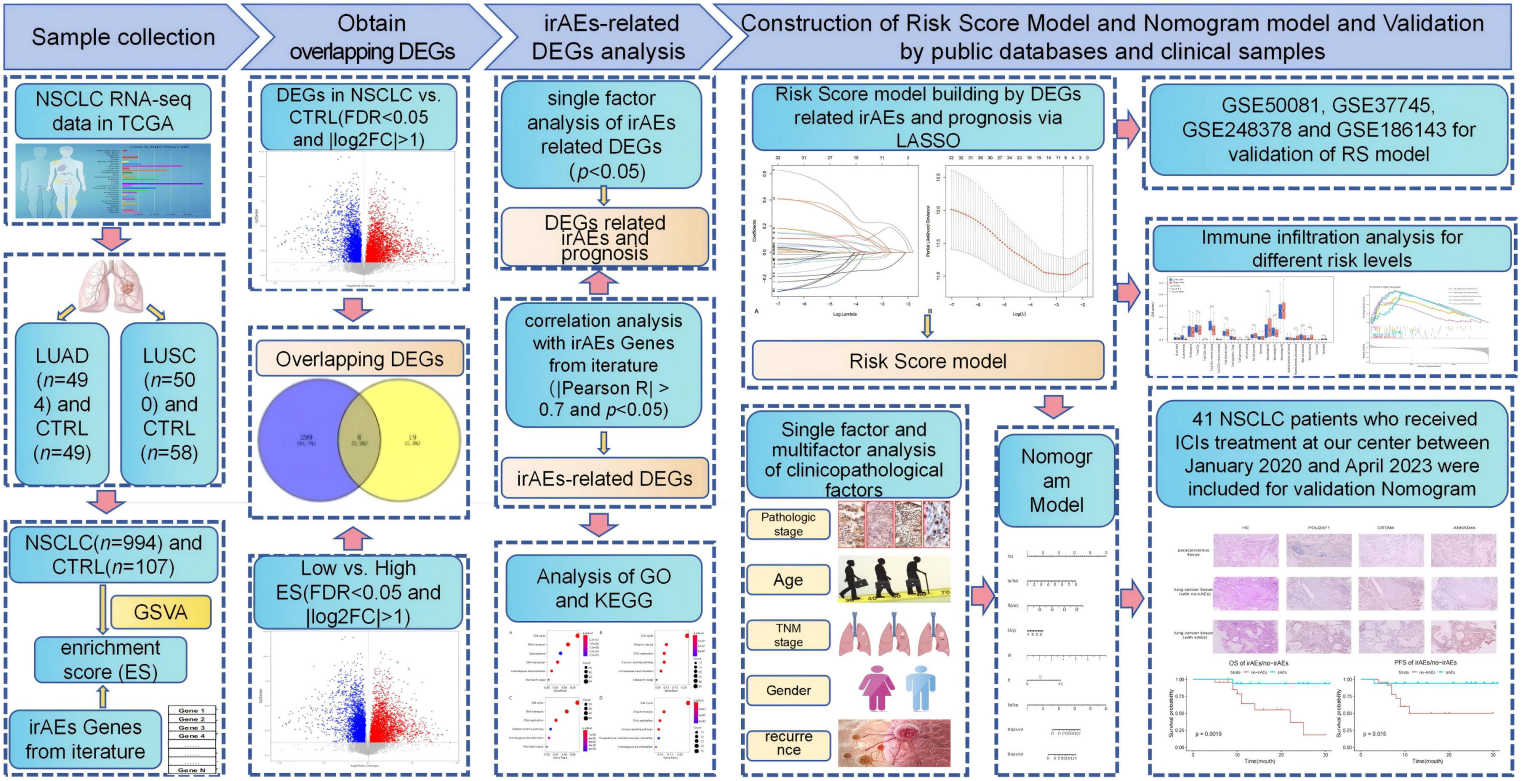
Schematic diagram of this study.

Our study design is illustrated in **Figure 1**. The gene expression data and clinicopathological parameters of lung adenocarcinoma (LUAD) and lung squamous cell carcinoma (LUSC) were downloaded from The Cancer Genome Atlas (TCGA) database (https://gdc-portal.nci.nih.gov/), including 994 cancerous and 107 normal samples with prognostic information. Additionally, gene expression patterns of lung cancer with prognostic information were also obtained from the Gene Expression Omnibus (GEO) database (https://www.ncbi.nlm.nih.gov/geo/), namely, GSE50081 (16), GSE37745 (17), and GSE248378 (18). Meanwhile, GSE186143 (19), an independent cohort of malignant melanoma receiving ICI treatment, was also downloaded for the subsequent study. All data were assessed using the Illumina HiSeq 2000 RNA Sequencing platform.

### 2.2 Screening of irAE-related DEGs

Gene expression data for LUAD (*n* = 585) and LUSC (*n* = 550) were downloaded from TCGA, generated on the Illumina HiSeq 2000 platform as standardized log_2_(FPKM + 1)values. After integrating clinical information, a total of 994 NSCLC tumor samples and 107 normal control samples with complete survival data were included in the final analysis (**Supplementary Tables 1, 2**). To leverage transcriptomic data reflecting immune dysregulation in irAEs—a strategy supported by prior studies—we prioritized TCGA-LC for analysis. This public dataset, with comprehensive lung cancer transcriptomes and clinical annotations, allowed cost-effective validation of gene expression in large cohorts. Firstly, we curated an initial set of irAE-related genes from the literature (20, 21) and applied objective statistical criteria [FDR < 0.05, *p* < 1 × 10^−4^ and RS (Spearman’s correlation coefficients) ≥0.7] to select the 30 most significant immunologic gene sets for further analysis (**Supplementary Table 3**), which represent key immune pathways in irAE pathogenesis. Subsequently, using GSVA (R v1.48.0), we assessed enrichment scores (ESs) for these gene sets in tumors *vs*. normal lung tissues to quantify their tumor microenvironment activity. Then, the optimal ES cutoff was determined via the X-tile software (Yale University, version 3.6.1) by maximizing the separation of high-/low-ES samples in TCGA-LC, as defined by the cutoff that generated the maximum log-rank *χ*² statistic. This cutoff was further validated by comparing overall survival between high- and low-ES groups. Next, differentially expressed genes (DEGs) were identified using Student’s *t*-test on the log_2_(FPKM + 1) normalized expression values, with adjustment for multiple testing using the Benjamini–Hochberg false discovery rate (FDR) procedure, with significance thresholds set at FDR <0.05 and |log_2_FC| >1. This analysis was performed between high- and low-ES specimens, as well as between cancer and non-cancer samples. By intersection analysis of DEGs between the above different groups, the correlations between the overlapping DEGs and the 30 genes related to irAEs were analyzed by applying the “cor” function in R. The genes with threshold values of |Pearson *R*| >0.7 and *p <*0.05 were identified as irAE-related DEGs. Then, to explore the biological significance and pathway enrichment, Gene Ontology (GO) analysis and Kyoto Encyclopedia of Genes and Genomes (KEGG) pathway analysis were performed using the Database for Annotation, Visualization, and Integrated Discovery (DAVID) (22).

### 2.3 RS model construction based on irAE-related DEGs

Based on TCGA-LC samples, we first identified prognosis-related genes from irAE-related DEGs using univariate Cox regression (R package survival) with a significance threshold of *p <*0.05. Next, we applied the least absolute shrinkage and selection operator (LASSO) regression (R package lars) to derive a parsimonious subset of prognostic genes, selecting the optimal regularization parameter (*λ*) via 10-fold cross-validation under the minimum-criteria rule. These candidates were then entered into a multivariate Cox regression to construct a multigene predictive model. The irAE-related prognostic risk score was calculated as follows:

RS=*Coef*_Gene1_×Gene1 expression+*Coef*_Gene2_×Gene2 expression+…+*Coef*_Gene n_ ×Gene n expression.

Where, *Coef*_Gene n_ is the regression coefficient of Gene n.

Using this expression, the RSs of samples in the TCGA lung cancer dataset and the GEO validation dataset were analyzed. According to the median of RSs, all specimens were divided into high- and low-risk groups in the training and validation datasets of lung cancer, and survival analysis was conducted to assess the prognosis between different risk groups via the “survival” package (*p <*0.05). Additionally, the differences of RS values between irAE and non-irAE groups in the GSE186143 dataset were analyzed via the Kruskal–Wallis rank sum test.

### 2.4 Immune microenvironment and immune escape assessment in different risk groups

Based on the expression levels in each sample of the TCGA-LC cohort, the proportion of immune cells in the tumor microenvironment was evaluated via the CIBERSORT algorithm, and the differences in immune cells (DICs) between high- and low-risk subtypes were analyzed via the “preprocessCore,” “e1071,” and “limma” packages. Additionally, the estimate package in R was applied to estimate the immune and stromal components via the ESIMATE scores, and the differences in stromal and immune scores between different risk groups were estimated using the “estimate” and “limma” packages. Furthermore, between the two risk groups, Hallmark signaling pathways were identified using gene set enrichment analysis (GSEA), which were analyzed by the “org.Hs.eg.db,” “clusterProfiler,” and “enrichplot” packages. Moreover, the expression of human leukocyte antigen (HLA) genes was analyzed via the “limma” package, with a threshold of *p <*0.05. The tumor immune dysfunction and exclusion (TIDE) scores of NSCLC samples were calculated utilizing the Tumor Immune Dysfunction and Exclusion (TIDE, http://tide.dfci.harvard.edu/login/) (23).

### 2.5 Nomogram model construction and evaluation

To study the independent prognostic factors, RSs and clinicopathological parameters in the TCGA-LC dataset were subjected to univariate and multivariate Cox regression analysis through the “survival” package, with a threshold value of *p <*0.05. Then, a nomogram model was constructed based on the independent parameters via the “rms” package. Concordance index (C-index) and area under the curve (AUC) for 1-, 3-, and 5-year survival probabilities were analyzed to evaluate the predictive accuracy of the nomogram model. Furthermore, to specifically assess the model’s stability and correct for overoptimism, we performed an internal validation using bootstrap resampling with 1,000 repetitions. The optimism-corrected C-index was calculated to evaluate the calibrated predictive performance.

### 2.6 Patients and treatments

A total of 41 patients with histologically confirmed NSCLC who were treated with ICIs were included from January 2020 to April 2023 at the 920^th^ Hospital (Yunnan, China). All data, including patient history, laboratory results, and irAEs, were obtained retrospectively. In our study, ICIs for NSCLC included sintilimab, camrelizumab, and tislelizumab, one of which was administered at a fixed dose every 3 weeks, as there was no evidence which ICI is superior to the others. All the patients were followed up for more than 6 months, and computed tomography (CT) was performed to evaluate the patients’ therapeutic response according to the Response Evaluation Criteria in Solid Tumors (RECIST) V1.1 (24). Furthermore, their irAEs were evaluated at every visit according to the Common Terminology Criteria for Adverse Events (CTCAE) version 5.0 (25). Two independent oncologists jointly determined irAEs based on the patients’ clinical symptoms, laboratory tests (e.g., liver function indicators, thyroid function), and imaging reports. In cases of disagreement in grading, a third expert was consulted for arbitration. irAEs were classified into grades 1–5 based on severity, with events of grade ≥3 defined as severe toxic reactions. The Institutional Ethics Committee at the 920^th^ Hospital approved this research, and informed consent based on the Declaration of Helsinki was obtained from all patients.

### 2.7 Immunohistochemical staining

The specimens from the patients were fixed in 10% formalin, dehydrated with a series of acetone, and embedded in paraffin. The specimens were sliced into 4-μm slices and analyzed by hematoxylin and eosin staining. Immunohistochemistry (IHC) was performed to assess the protein expression of CRTAM, ANKRD44, and POU2AF1 on formalin-fixed, paraffin-embedded (FFPE) tissue sections. The following primary antibodies were used: rabbit monoclonal anti-CRTAM (Cat. ab820487, Zen bioscience, Chengdu, China; dilution 1:100), rabbit polyclonal anti-ANKRD44 (Cat. AP50195PU-N, OriGene, Beijing, China; dilution 1:200), and rabbit recombinant monoclonal anti-POU2AF1 (Cat. ab76010, Hua’an Biotechnology, Hangzhou, China; dilution 1:200). Antigen retrieval was performed in citrate buffer (pH 6.0) at 95°C for 20 min. Sections were then incubated with primary antibodies overnight at 4°C. After washing, immunostaining was visualized using a DAB detection system and counterstained with hematoxylin. Two senior pathologists reviewed the slides independently. The intensity scoring criteria were as follows: 0, no staining; 1, <10% staining; 2, 10% to 50% staining; and 3, 50% to 100% staining. The final IHC score was the sum of both scores: 0–2 for negative and 3–6 for positive.

### 2.8 Statistical analysis

All data processing was performed using R version 3.6.1. The normality of continuous variables was assessed using the Shapiro–Wilk test. Based on the results of the normality test and the exploratory nature of the data, non-parametric methods were primarily employed for group comparisons. Continuous variables were analyzed using the Kruskal–Wallis test and the Wilcoxon rank-sum test, while categorical variables were compared using the chi-square (*χ*²) test. Spearman’s correlation test was applied to determine correlation coefficients. Independent predictive factors were identified through Cox proportional hazards regression analysis. Statistical significance was defined as *p <*0.05.

## 3 Results

### 3.1 Construction of RS for NSCLC

**Figure 2:**
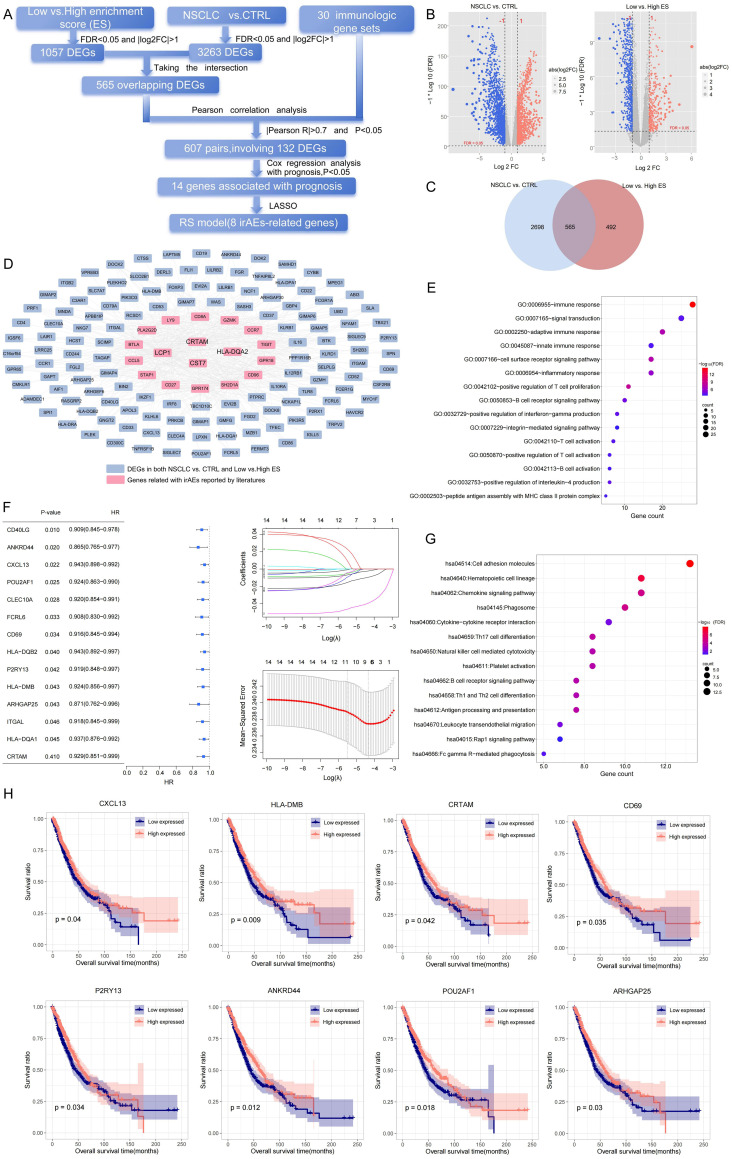
Construction of risk scores for NSCLC. **(A)** The screening process of irAE-related DEGs. **(B)** DEGs in the NSCLC *vs*. normal groups (left) and the low- *vs*. high-ES groups (right). **(C)** Overlapping DEGs between the low- *vs*. high-ES groups and the NSCLC *vs*. normal groups. **(D)** Correlation network of 30 genes reported by the literature and 132 irAE-related DEGs. **(F)** Univariate Cox analysis (left) and LASSO parameter plots (right) of 14 irAE-related genes. **(E, G)** GO enrichment analysis **(E)** and KEGG signaling pathway analysis associated with irAE-related DEGs **(G)**. **(H)** KM plots of eight irAE-related genes. Blue and red curves represent low- and high-expression subgroups, respectively.

The screening strategy of irAE-related DEGs in our study is shown in **Figure 2A**. GSVA was conducted to assess the ESs of 30 immunologic gene sets reported in the literature based on the TCGA-LC dataset. Normal lung tissues exhibited significantly higher ES values than NSCLC samples (**Supplementary Figure S1A**, *p* < 0.05), confirming immune pathway suppression in tumors. According to the optimal cutoff value of 0.23, the lung cancer samples were divided into high-ES groups (*n* = 761) and low-ES groups (*n* = 233). Notably, the OS of the high-ES group was significantly longer than that of the low-ES group (*p* < 0.05) (**Supplementary Figure S1B**). Then, 1,057 DEGs between the high- and low-ES groups were identified. After intersection with 3,263 DEGs between cancer and normal groups, 565 overlapping DEGs were obtained (**Figures 2B, C**; **Supplementary Table 4**). By conducting further Pearson correlation analysis, 132 irAE-related DEGs were identified (**Figure 2D**), with a total of 607 pairs of connections (|Pearson *R*| > 0.7 and *p* < 0.05, **Supplementary Table 5**).

To elucidate the biological significance and pathway enrichment underlying the irAE-related DEG set, GO and KEGG pathway analyses were conducted, respectively. As shown by GO analysis, 50 biological processes, including cytokine activation, cell adhesion, and receptor recognition, were significantly enriched. Meanwhile, some immunologic regulatory pathways, such as immune response, inflammatory response, and positive regulation of T-cell proliferation, were identified via KEGG analysis (**Figures 2F, G**; **Supplementary Table 6**).

Moreover, according to the prognostic information in the TCGA-LC dataset, 14 prognosis-related genes were identified from 132 irAE-related DEGs. To avoid overfitting, LASSO regression analysis was further performed. A panel of eight feature genes was selected and used to generate the RS model as follows (**Figure 2E**):

RS = (−0.0443) × ANKRD44 + (−0.0134) × POU2AF1 + (−0.0056) × P2RY13 + (−0.0056) × CXCL13 + (−0.0036) × CD69 + (−0.0025) × HLA-DMB + (−0.0002) × ARHGAP25 + (−0.0160) × CRTAM

Furthermore, the TCGA-LC training dataset was clustered into different subtypes based on the medium expression of each feature gene. Kaplan–Meier (KM) survival analysis revealed that there was a significant difference in prognosis between the high and low subtypes (**Figure 2H**, p < 0.05).

### 3.2 Validation of the prognostic value of RS

**Figure 3:**
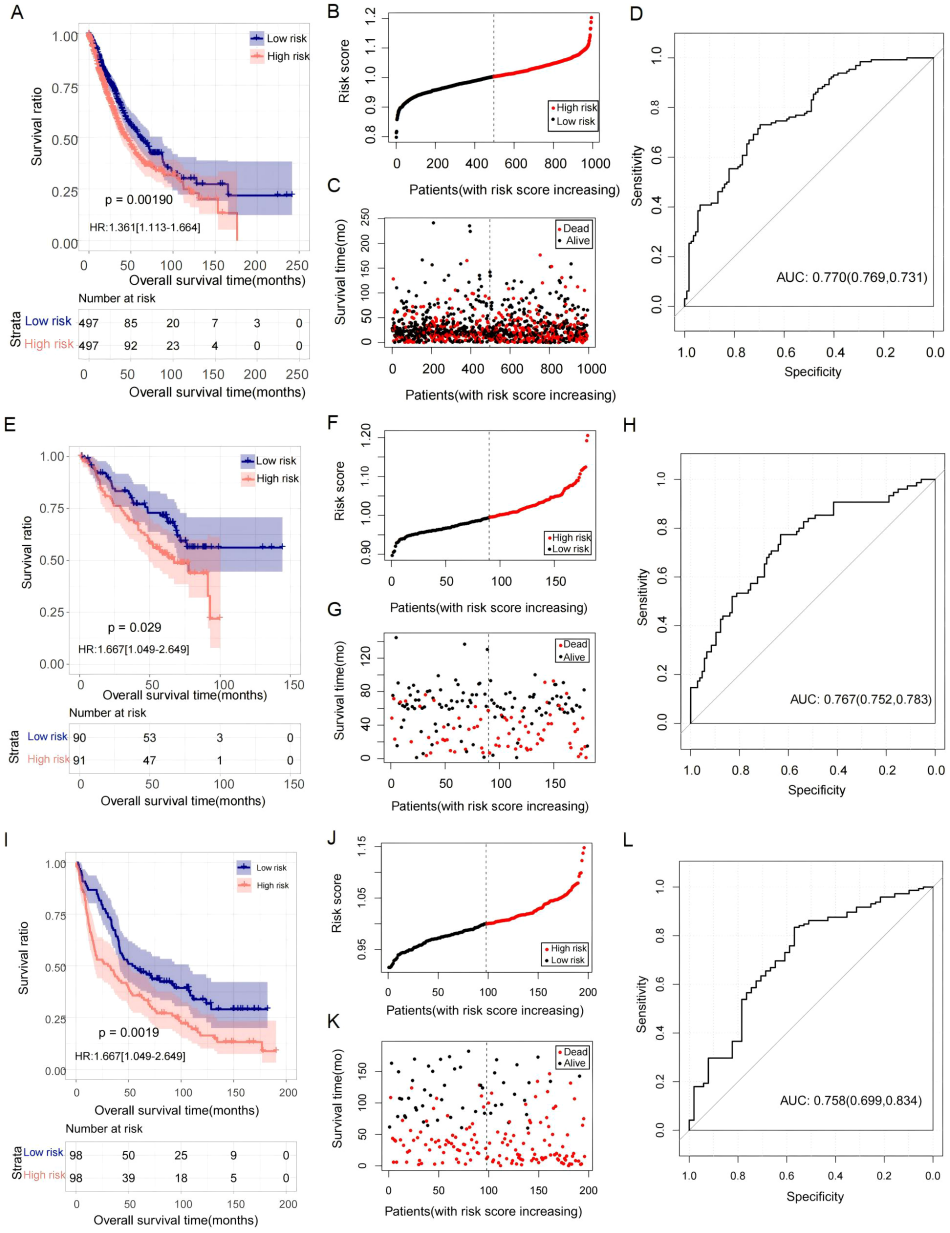
The prognostic value of the RS models. **(A–D)** KM curves, RS distribution, survival status, and ROC curve in the TCGA cohort, respectively. **(E–H)** KM curves, RS distribution, survival status, and ROC curve in GSE50081, respectively. **(I–L)** KM curves, RS distribution, survival status, and ROC curve in GSE37745, respectively. For KM curves, the blue and red curves represent the survival ratio in the low- and high-RS groups, respectively.

The RS of each sample was analyzed in the TCGA lung cancer dataset. According to the median level of RS, patients were stratified into high- and low-risk groups. Compared to the TCGA lung cancer cases in the low-risk group, the OS rate in the high-risk group was shorter (**Figure 3A**). ROC analysis showed that the predictive value (AUC) of this RS for the 5-year survival rates of lung cancer patients in the TCGA dataset was 0.770 (**Figure 3D**). We also observed that the proportion of deaths in the high-risk group was higher than in the low-risk group (**Figures 3B, C**). Furthermore, the prognostic value of the RS was also validated in the GSE50081 and GSE37745 datasets. In these two cohorts, the OS rates of patients with high RSs were significantly lower than those with low RSs (**Figures 3E, I**), and the AUC values for predicting the 5-year survival rate were 0.767 and 0.758, respectively (**Figures 3H, L**). Meanwhile, a higher proportion of deaths was also identified in the high-risk group (**Figures 3F, G, J, K**).

### 3.3 Predictive value of immunotherapy response

**Figure 4:**
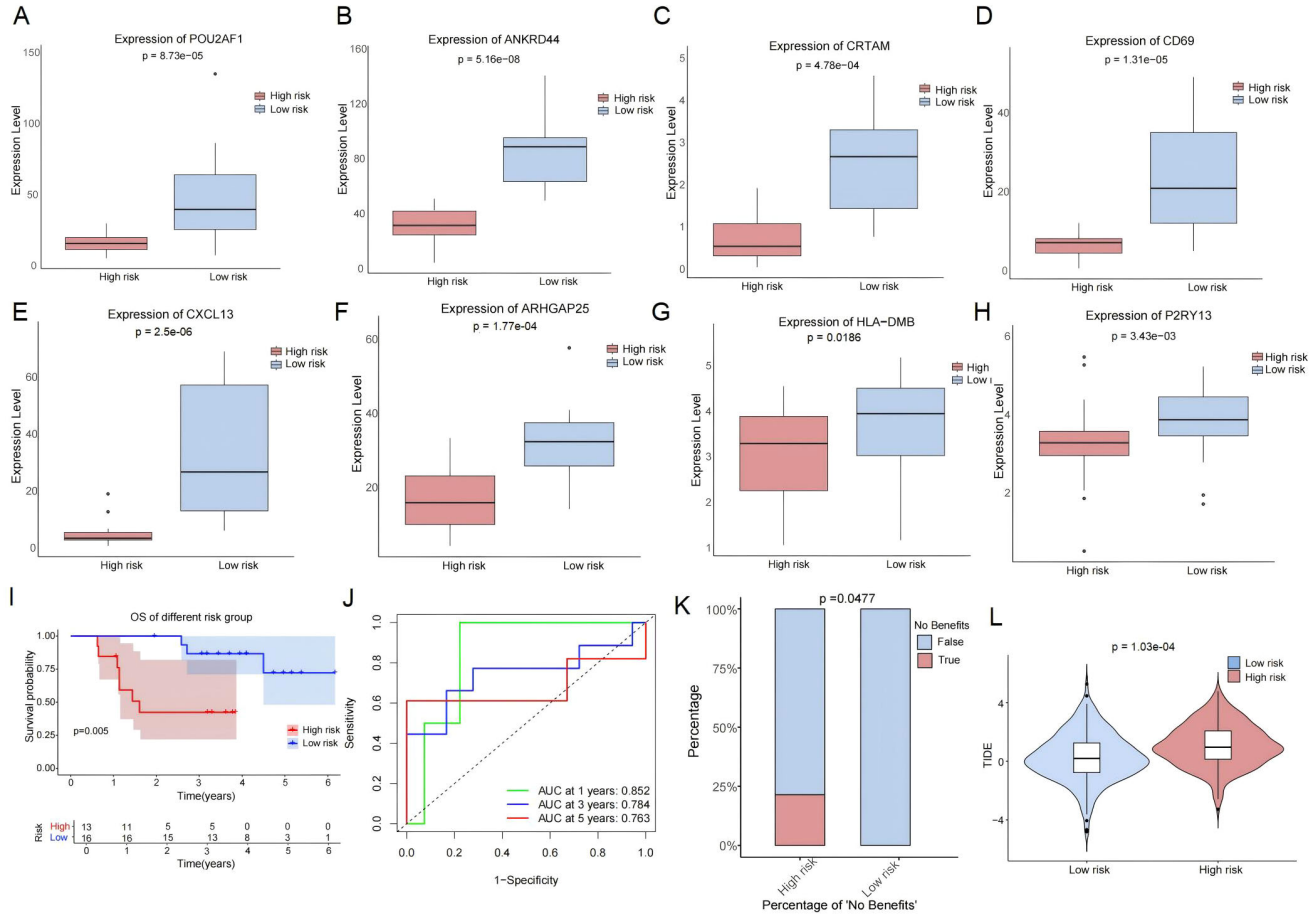
Predictive value of RS for immunotherapy response in GSE248378. **(A–H)** Differential expressions of eight signature genes in different risk groups. **(I, J)** Kaplan–Meier **(I)** and time-dependent ROC **(J)** curves in different risk groups. **(K)** The proportion of patients with no benefits in different risk groups. **(L)** The TIDE scores in different risk groups.

To investigate the ability of RS to predict immunotherapy efficacy, RS was applied to the GSE248378 cohort, in which all lung cancer patients had complete clinical information of ICI treatment. Firstly, the expression of eight signature genes of RS (ANKRD44, POU2AF1, P2RY13, CXCL13, CD69, HLA-DMB, ARHGAP25, and CRTAM) was investigated between the two RS groups. In the low-RS group, eight feature genes were upregulated (**Figures 4A–H**). When compared to the high-risk group, the patients in the low-risk group had significantly better OS (**Figure 4I**). Time-dependent ROC analysis showed that the AUC values at 1, 3, and 5 years for predicting OS associated with ICI treatment were 0.852, 0.784, and 0.763, respectively (**Figures 4J**). The proportion of patients with no benefits was lower in the low-risk group than in the high-risk group (**Figure 4K**). Moreover, by TIDE analysis, we found that the patients with low RSs had lower TIDE scores than those with high RSs (**Figure 4L**). These results indicated that patients in the high-risk group exhibited resistance to ICIs.

### 3.4 Predictive value of irAEs

**Figure 5:**
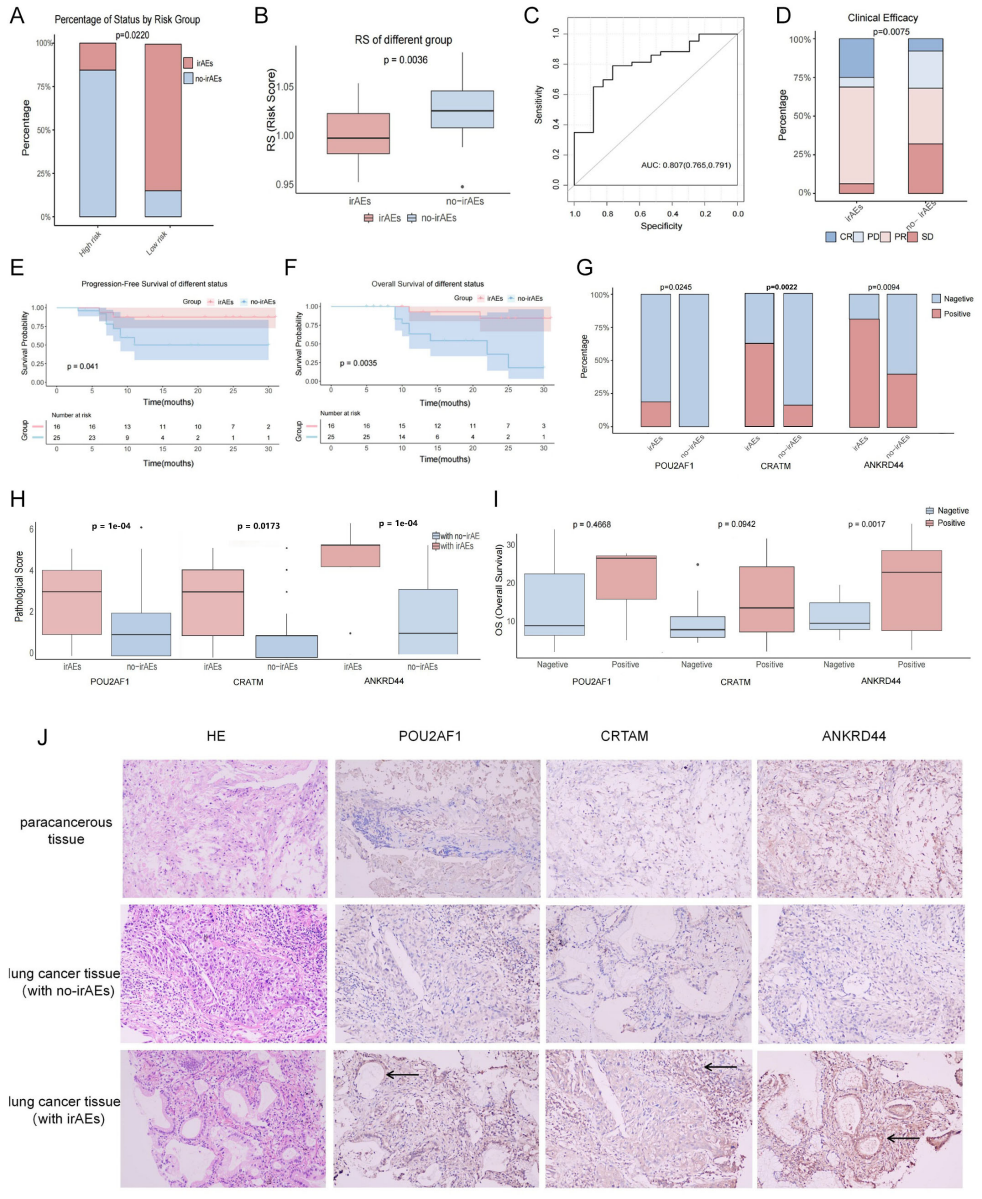
Predictive value of irAEs in GSE186143 and 41 NSCLC patients. **(A–C)** GSE186143. The incidence of irAEs in the low- and high-RS groups **(A)**; RS differences between the irAE and no-irAE groups **(B)**, with blue and red curves representing the no-irAE and irAE groups, respectively. **(C)** ROC curves predicting irAE occurrence, with numbers in parentheses indicating specificity and sensitivity. **(D)** Distribution of clinical efficacy (CR, PR, SD, and PD) after ICI treatment in patients with irAEs and no-irAEs. **(E, F)** The OS **(E)** and PFS **(F)** of the irAE group and the no-irAE group. **(G–I)** Expression of the three risk genes [POU2AF1 **(G)**, CRTAM **(H)**, ANKRD44 **(I)**] in patients with no-irAEs and irAEs **(G)**, protein level **(H)**, and OS **(I)** of negative and positive patients. **(J)** Expression differences of the three key model genes (POU2AF1, CRTAM, ANKRD44) in patients with lung cancer adjacent tissues, lung tumor tissues with irAEs, and lung tumor tissues with no-irAEs (image magnification of 10 × 20). Black arrows indicate areas of significant immunohistochemical positivity.

Considering that the RS was conducted based on irAE-related genes, we evaluated the predictive value of irAEs. In the melanoma cohort GSE186143 with clinical information of irAEs, we found that the incidence of irAEs in low-risk group was significantly higher than in the high-risk group, and patients with irAEs had significantly lower RS than patients without irAEs (**Figures 5A, B**). Further analysis revealed that the AUC value of the RS to predict irAEs was 0.807 (**Figure 5C**). In addition, we investigated the potential of irAE prediction in our cohort with a total of 41 NSCLC patients with ICI treatment (**Supplementary Table 7**). Among them, 16 patients (39.02%) experienced at least one adverse event, and 25 patients (60.98%) had no irAEs. The most common irAEs were thyroiditis (*n* = 5, 31.25%), followed by reactive cutaneous capillary endothelial proliferation (RCCEP) (*n* = 4, 25%) and immune-related pneumonia (*n* = 2, 12.5%). Among the patients with irAEs, 4 patients (25%) presented with a complete response (CR), 10 patients (62.5%) developed a partial response (PR), 1 patient (6.25%) had stable disease (SD), and 1 patient (6.25%) had progressive disease (PD). Meanwhile, in the non-irAE group, the number of patients with CR, PR, SD, and PD was 2, 9, 8, and 6, respectively (**Supplementary Table 8**). The objective response rate (ORR, CR+PR) and the disease control rate (DCR, CR+PR+SD) were higher in the irAE group than in the non-irAE group (87.50% *vs*. 44.00%, 93.75% *vs*. 76.00%) (**Figure 5D**). Furthermore, patients with irAEs had a significantly longer PFS and OS than patients without irAEs (median PFS: 20.00 *vs*. 9.86 months; median OS: 21.37 *vs*. 12.08 months) (**Figures 5E, F**). Next, we evaluated the expressions of three risk genes with the highest weights (POU2AF1, ANKRD44, and CRTAM) in patients with irAEs versus those without irAEs. As compared to patients without irAEs, the protein level and positive rate of CRTAM, POU2AF1, and ANKRD44 were increased in patients with irAEs (**Figures 5G, H**). We further evaluated the correlation between survival period and protein expression and found that the OS in the ANKRD44-positive group was significantly improved than in the negative group (**Figure 5I**). The expression of POU2AF1, CRTAM, and ANKRD44 was significantly upregulated in lung tumor tissues with irAEs (10.5-fold, 1.97-fold, and 2.62-fold *vs*. no irAEs, *p* < 0.05) and showed a positive correlation with irAE occurrence (**Supplementary Table 9**). Immunohistochemical staining (10 × 20 magnification) further validated nuclear localization differences in the three risk gene expression patterns (**Figure 5J**).

### 3.5 Immune analysis related to the RS

**Figure 6:**
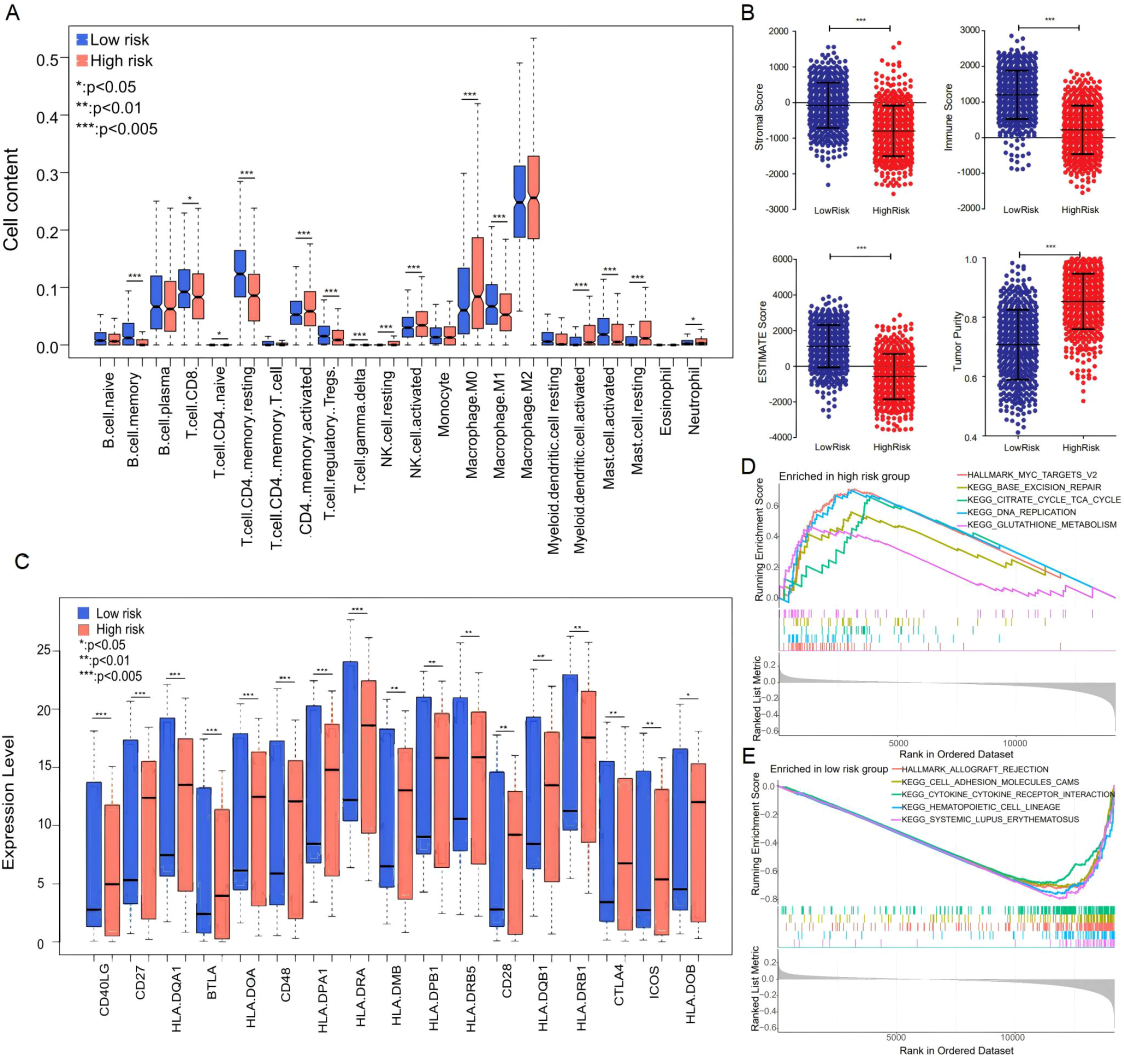
Immune analysis related to the RS. **(A)** Distribution of various immune cell types in different risk groups. **(B)** Stromal score, immune score, ESTIMATE score, and tumor purity in different risk groups. **(C)** Expression levels of HLA genes in different risk groups. **(D, E)** Hallmark signaling pathways significantly correlated with the low- **(D)** and high-risk **(E)** groups. * denotes p < 0.05, ** denotes p < 0.01, *** denotes p < 0.001.

Because dysregulation of immune cell infiltration in the TME plays a crucial role in immunotherapy, we investigated the correlation between immune cell infiltration and RS based on the CIBERSORT algorithms. The proportion of 22 types of immune cells was quantified on the expression data in TCGA-LC samples. As compared with the high-risk group, the abundance of CD8^+^ T cells, CD4^+^ memory resting T cells, M1 macrophages, and activated mast cells was obviously elevated in the low-risk group (**Figure 6A**). Based on the estimate algorithm, we found that the stromal, immune, and ESTIMATE scores were significantly higher in the low-risk group, along with a dramatic decrease in tumor purity (**Figure 6B**). We further used GSEA to search for Hallmark terms across the TCGA-LC dataset and found that the significantly enriched Hallmark terms in the low-risk group were cytokine–cytokine receptor interaction, cell adhesion molecules, etc. (**Figures 6D, E**). Meanwhile, the differences of human leukocyte antigen (HLA) genes were assessed between the two risk groups, which were essential in antigen presentation. As compared with the high-risk group, most HLA genes, such as HLA-I and HLA-II, had higher expression levels in the low-risk group (**Figure 6C**). Our results indicated that risk score was closely related to TME in lung cancer patients, in which the low-risk group tended to have more immune cells with enhanced antitumor immune responses.

### 3.6 A nomogram based on RS and clinicopathology parameters

**Figure 7:**
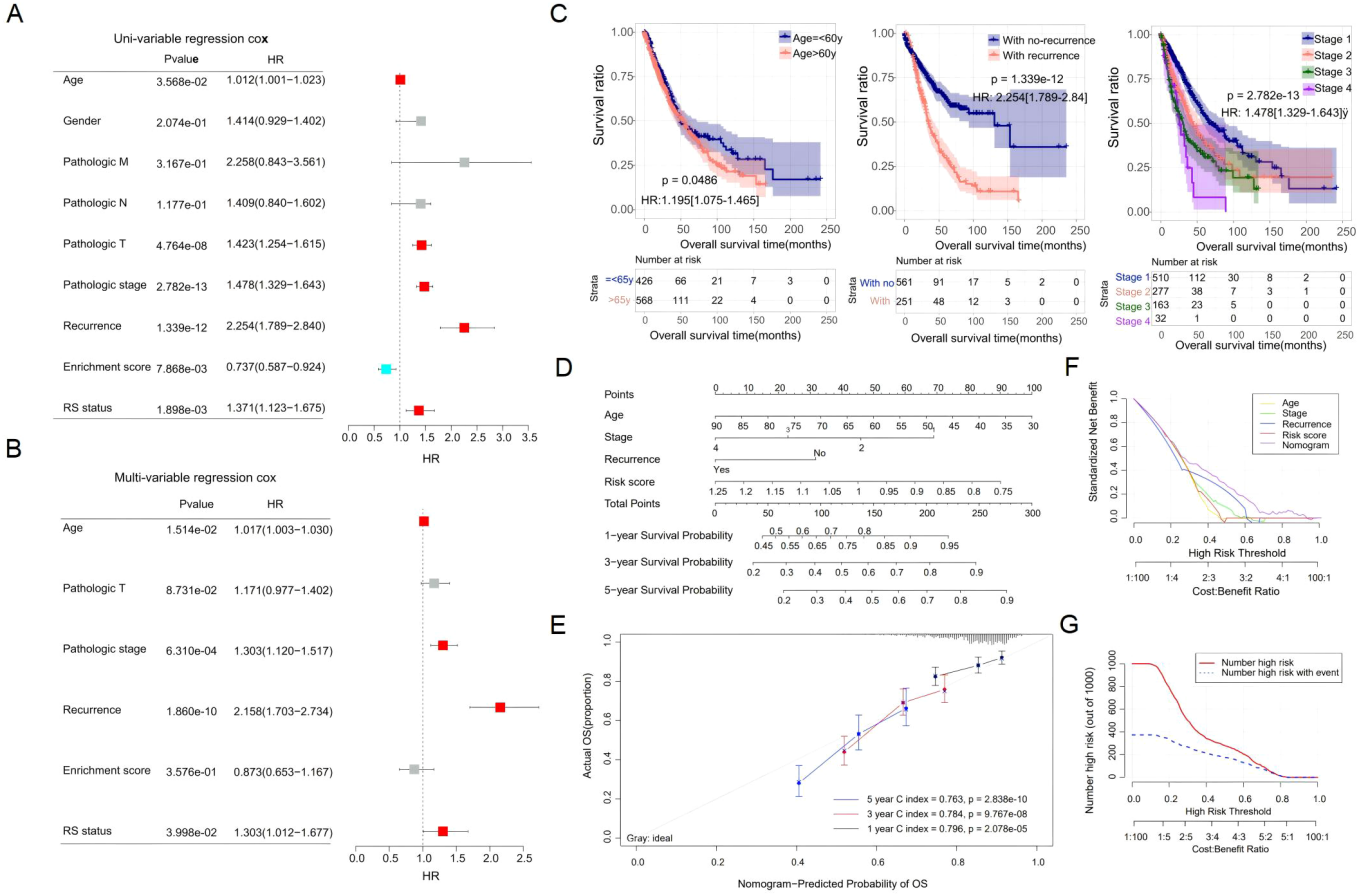
Construction of the nomogram model based on multi-omics. **(A, B)** Forest plots of univariate **(A)** and multivariate **(B)** Cox regression for clinical parameters and RS. **(C)** KM plots for different subgroups of age (left), recurrence (middle), and stage (right). **(D)** Nomogram plot for the survival prediction model based on independent prognostic factors. **(E)** Consistency between predicted and actual survival rates at 1, 3, and 5 years, with the horizontal axis indicating predicted survival rates and the vertical axis indicating actual survival rates. **(F, G)** Decision line analysis curve plot [decision analysis curve **(F)** and clinical impact curve **(G)**].

To explore the independent prognostic factors for patients with lung cancer, we combined clinical features and the precalculated score value into an integrated analysis. As shown by the univariate Cox regression analysis, some factors, including age, pathological T, clinical stage, tumor recurrence, enrichment score of irAEs, and RS, were significantly related to OS (*p* < 0.05) (**Figure 7A**). The multivariate Cox regression analysis further revealed that age, clinical stage, tumor recurrence, and RS were independent prognostic factors (*p* < 0.05) (**Figure 7B**). The detailed statistical results are shown in **Supplementary Table 10**. The effect of age, clinical stage, and tumor recurrence on survival was shown by KM plot analysis (**Figure 7C**). To improve the clinical unity of RS, a nomogram was established based on the four independent factors (**Figure 7D**). The C-index values of our model at 1, 3, and 5 years were 0.796, 0.784, and 0.763, respectively (**Figure 7E**). As shown by the decision curve analysis (**Figure 7F**), the nomogram combined with four factors had a better clinical benefit net than that combined with a single factor. In addition, **Figure 7G** reflects the clinical benefit of the predictive model at different decision thresholds, indicating that the model has significant clinical value in identifying high-risk patients and guiding early intervention. Finally, bootstrap internal validation (*n* = 1,000) confirmed the model’s robustness. Visual comparison of the corrected calibration curves (**Supplementary Figure S2**) showed that our RS model’s performance surpassed all alternative predictors, highlighting its superior and stable predictive ability.

## 4 Discussion

Immunotherapy is one of the most important treatments for patients with advanced NSCLC, while only a fraction of patients have experienced long-term benefit from such treatments (26). Therefore, improved models urgently needed to stratify patients are a prerequisite for precision cancer medicine. Accumulated evidence has identified irAEs to be closely related to the therapeutic effects of ICIs (14, 27–29). Although prognostic scoring systems have been used to predict the outcome of cancer patients, there have been no studies that focused on the irAE gene-related prognostic signature in NSCLC.

Current immunotherapeutic biomarkers, including PD-L1 expression (TPS ≥ 50%) and tumor mutational burden (TMB-high, ≥10 mut/Mb), exhibit considerable limitations that constrain their predictive reliability. While associated with improved responses to ICIs, their efficacy is substantially undermined by tumor spatiotemporal heterogeneity; single-biopsy response rates in heterogeneous tumors can be as low as 15%–20%, and the concordance between PD-L1 and TMB positivity is only 54.8% (30). Although tertiary lymphoid structure (TLS) signatures offer an alternative for PD-L1-negative cases, their predictive accuracy remains unstable. Combination strategies have been explored to improve predictive power, yet they introduce new complexities. For instance, the co-occurrence of high TMB and high PD-L1 expression (TPS ≥ 50%) was associated with an objective response rate of up to 57% in patients treated with anti-PD-1 therapy (31). Similarly, the combination of an interferon-gamma (IFN-γ) gene signature with TMB identified patient subgroups with pathological response rates (pRR) ranging from 39% to 100% (32). However, their clinical translation is hindered by technical and biological complexities, as well as the low prevalence of dual-positive patients, limiting broad applicability. Notably, established benchmarks such as TMB and TIDE show AUC values ranging from 0.62 to 0.75 in predicting sustained clinical benefits or responses to immunotherapy, underscoring an unmet need for more robust predictors (33, 34). Against this backdrop, we developed a novel RS based on eight irAE-related genes, which effectively stratified patients into distinct risk groups with significantly divergent OS. The prognostic robustness of the RS was consistently validated across external datasets (GSE50081 and GSE37745). Our RS model demonstrated comparatively higher predictive performance, with AUCs of 0.852, 0.784, and 0.763 for 1-, 3-, and 5-year ICI-related OS predictions. This suggests that our model may provide complementary value to existing benchmarks like TMB and TIDE. Low-RS patients derived greater benefit from ICIs, showing alignment with and potential superiority to TIDE analysis, a computational framework known to outperform single biomarkers (23). This was further supported by significantly lower TIDE scores (indicative of immune escape and poor response) in the low-RS group, confirming the model’s capacity to capture critical tumor immune microenvironment dynamics. To enhance clinical applicability, we integrated the RS with key clinical features into a nomogram, which demonstrated higher predictive accuracy (C-index) and stability than the RS alone. By integrating clinical and molecular data, our model overcomes key limitations of single biomarkers, providing a cost-effective and reliable tool for personalized NSCLC immunotherapy where traditional biomarkers fall short.

Moreover, our RS presented a promising prediction effect of irAE incidence in a melanoma cohort. To further test its value in NSCLC, we validated the expression of RS genes with the top 3 genes ranked by regression coefficient. POU2AF1, a B-cell-specific transcriptional co-activator, regulates immune response via interaction with the octamer-binding transcription factors Oct-1 and Oct-2 (35). POU2AF1 is involved in promoting CD4^+^ T-cell memory and prospectively identifies memory precursors, which could improve the durable antitumor immune response (36, 37). CRTAM, a class I-restricted T-cell-associated molecule, is a cell adhesion molecule expressed by activated cytotoxic lymphocytes (38, 39). CRTAM–Necl-2 interactions promote cytotoxicity of NK cells and interferon-gamma secretion of CD8^+^ T cells in lung cancer, thus exerting the immunosurveillance role to distinguish tumors from normal cells (40). CRTAM also promotes antitumor immune response by enhanced CD8^+^ T-cell infiltration in breast cancer, which may correlate with the increased expression of MHC class I molecules (41). The ANKRD44 gene encodes for ankyrin repeat domain proteins, a member of a putative regulatory ARS subunit of the protein phosphatase 6 (PP6) complex (42, 43). ANKRD44 gene silencing constitutively activates the NF-kb protein via the TAK/AKT pathway, which leads to trastuzumab resistance via cell proliferation and glycolysis in breast cancer cells (44). ANKRD44 is also involved in immune cell metabolism and inflammation regulation via the mTOR pathway (45–47). In our NSCLC cohort, the presence of irAEs was mostly relevant to the efficacy of ICIs as well as improved survival. In line with the expression levels in the low-RS group, the increased expression of POU2AF1, CRTAM, and ANKRD44 was observed in the patients with irAEs, suggesting the potential of RS to predict irAEs in NSCLC. It is important to note that the mechanistic roles of POU2AF1, CRTAM, and ANKRD44 in irAEs are inferred from prior literature and clinical associations. While these inferences provide a rationale for the predictive capacity of our RS model, they remain hypothetical without direct functional validation in this study. The precise molecular pathways by which these genes influence irAE development thus require further investigation. Future studies using *in vitro* and *in vivo* models are essential to definitively establish their causal roles and underlying mechanisms in irAEs.

Due to the fact that immunological characteristics were associated with immune activity and immunotherapy efficacy, we explored the individual heterogeneity in the immune microenvironment. In our study, the abundance of cancer-suppressive immune cells was significantly decreased in patients with high RSs, accompanied by an increase in cancer-promoting immune cells. Moreover, we found that the risk score was highly negatively associated with immune and stromal scores while showing a positive association with tumor purity. In addition, the downregulated expression of most HLA genes was found in the high-risk group, which could prevent T cells from being activated to effectively kill tumors due to the lack of antigen presentation. Therefore, we speculated that the worse survival condition and therapeutic response may be due to the formation of an immune-suppressive microenvironment in patients with high RSs. However, this study has several limitations. First, our findings were obtained from public databases through retrospective analysis; thus, multicenter study cohorts still need to be used to validate the accuracy and stability of the RS. Second, we only validated the expression of the top 3 risk genes with high weight in our NSCLC cohort via IHC, and *in vitro* and *in vivo* assays designed to explore the functions and the underlying molecular mechanism need to be performed in a follow-up study. Third, while we integrated transcriptomics with clinical parameters to build the nomogram model, this transcriptomics-centric approach precluded the exploration of post-transcriptional, epigenetic, or proteomic mechanisms underlying irAE pathogenesis. Finally, the validation cohorts were primarily composed of Asian populations, which may limit the generalizability of our findings to other ethnic groups, such as those of African and European ancestry. Future work should incorporate multi-omics data to comprehensively dissect the molecular drivers of irAEs and refine the model’s mechanistic depth.

Overall, the risk signature constructed by eight key genes associated with irAEs had remarkable prognostic value, and the RS could accurately predict the immunotherapy response and immune-related adverse events, thus potentially guiding treatment choice for patients with NSCLC. Moreover, a nomogram model was developed to improve the prognostic stability in NSCLC. Our study may provide new ideas on the role of irAEs in the therapy of NSCLC.

## Data Availability

The original contributions presented in the study are included in the article/**Supplementary Material**. Further inquiries can be directed to the corresponding authors.
