# Supplementary figures and images for: Identification of a prognostic signature based on immunogenic adverse event-related genes to guide therapy for non-small cell lung cancer
Source: Front Immunol. 2026 Jan 12;16:1656375. doi: 10.3389/fimmu.2025.1656375 (PMC12833344; doi:10.3389/fimmu.2025.1656375)

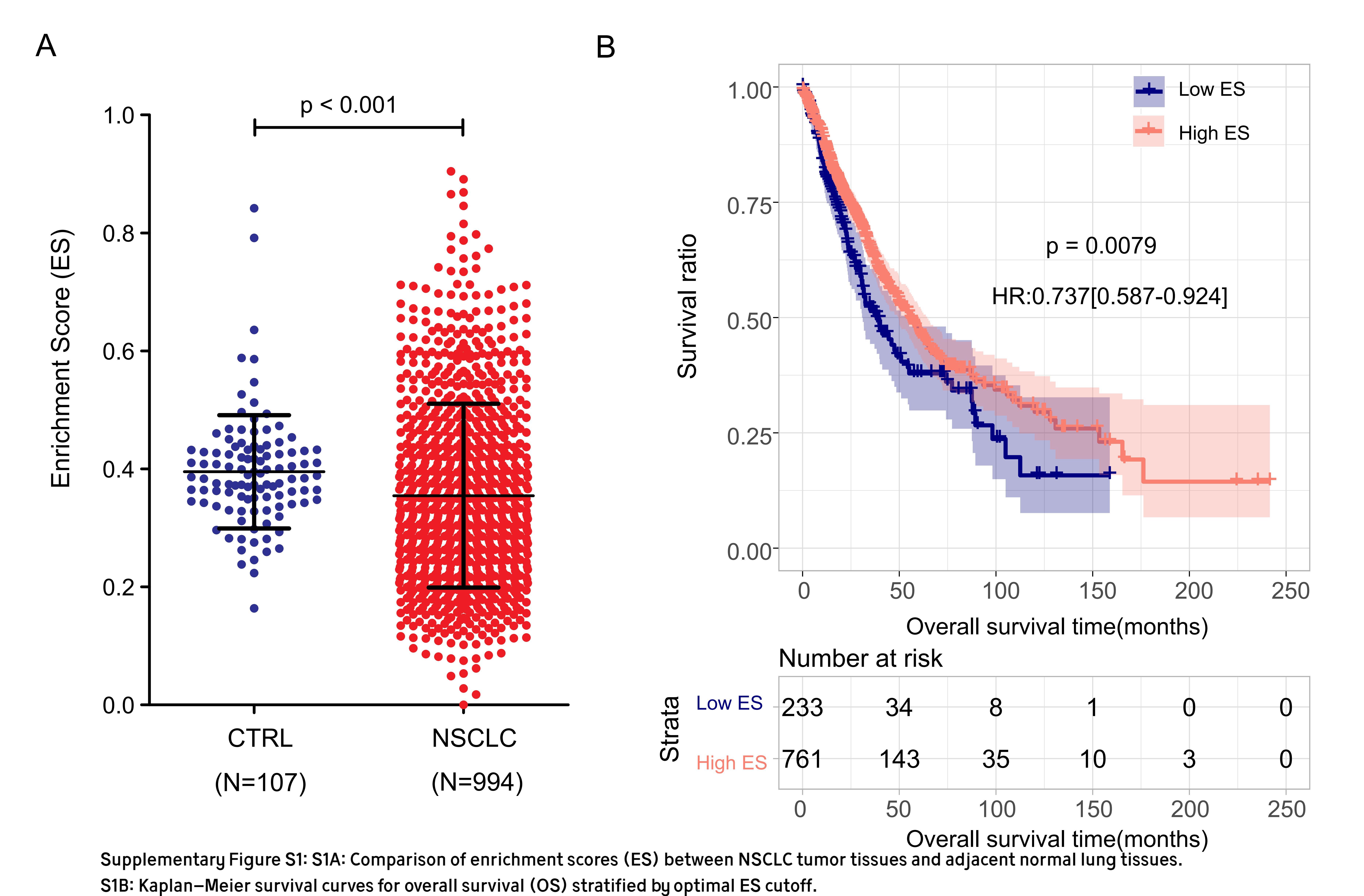

Supplement: Supplementary file 1 [file Image1.jpeg]

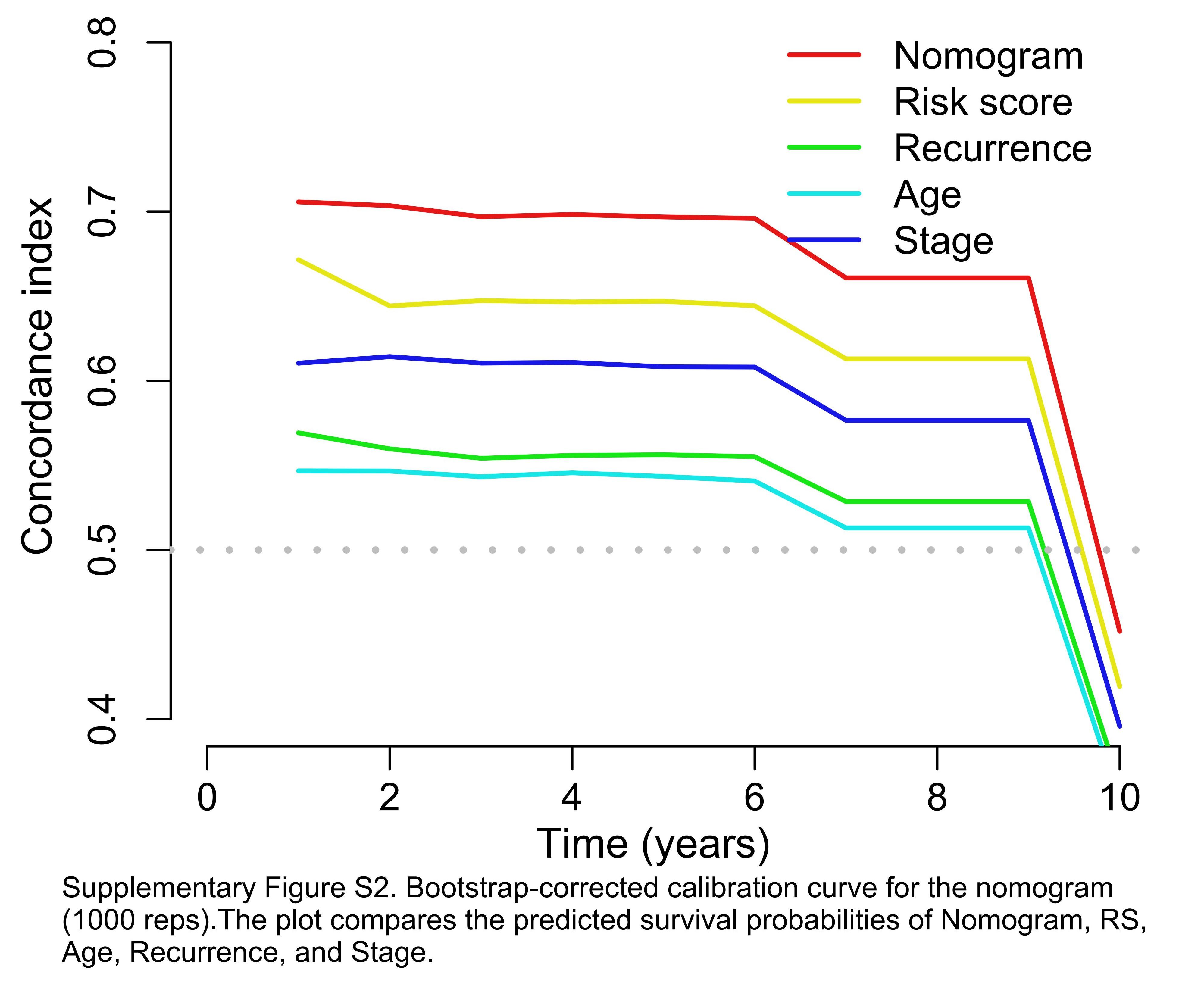

Supplement: Supplementary file 2 [file Image2.jpeg]
